# Intrasinusoidal pattern of bone marrow infiltration by hepatosplenic T‐cell lymphoma

**DOI:** 10.1002/ccr3.1423

**Published:** 2018-02-13

**Authors:** Liesl Ann Butler, Surender Juneja

**Affiliations:** ^1^ Victorian Comprehensive Cancer Centre 305 Grattan Street Melbourne Victoria 3000 Australia; ^2^ Peter MacCallum Cancer Centre 305 Grattan Street Melbourne Victoria 3000 Australia; ^3^ Royal Melbourne Hospital 300 Grattan Street Parkville Victoria 3050 Australia; ^4^ Department of Pathology University of Melbourne Building 181, Grattan Street Parkville Victoria 3052 Australia

**Keywords:** Hepatosplenic, intrasinusoidal, lymphoma, non‐hodgkin, T‐cell

## Abstract

Hepatosplenic T‐cell lymphoma is a rare, aggressive form of extranodal lymphoma, which frequently involves the bone marrow. An intrasinusoidal pattern of infiltration is characteristic of the disease and is often best appreciated on immunohistochemical staining. Bone marrow biopsy can be a useful diagnostic tool.

A 51‐year‐old man presented with pancytopenia, constitutional symptoms, and splenomegaly. His past history was significant for hepatosplenic T‐cell lymphoma, from which he had achieved a complete remission (five‐year duration) following autologous stem cell transplant.

His blood film was leucoerythroblastic, while positron emission tomography (PET) was suspicious for relapsed disease.

Bone marrow biopsy demonstrated a mild lymphoid infiltrate in a predominately sinusoidal distribution (Figs 1 and 2). Cells were CD3‐positive, but negative for CD4 and CD8 on immunohistochemistry. Given adequate trilineage hematopoiesis, the cytopenias were considered to be due to increased peripheral destruction/sequestration. The abnormal lymphoid population was confirmed by immunophenotyping. Cells expressed CD2, CD3, CD7, CD16/56, CD94, and TCR‐gamma/delta; they were CD4, CD5, CD8, CD26, and CD27 negative.

**Figure 1:**
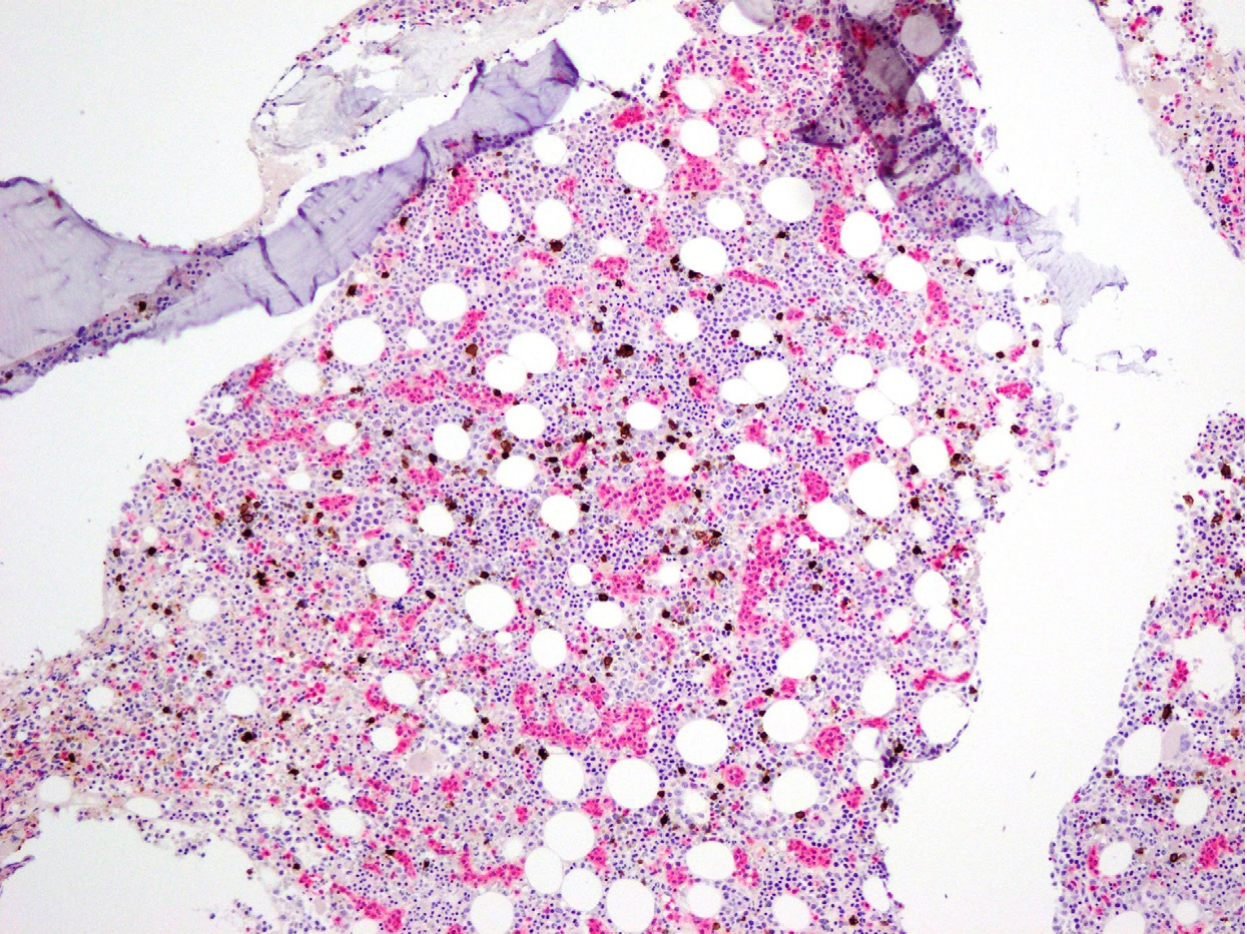
Illustrates the classical intrasinusoidal pattern of marrow involvement on immunohistochemistry using a dual CD3 (pink) and CD20 (brown) stain at x10 magnification.

**Figure 2:**
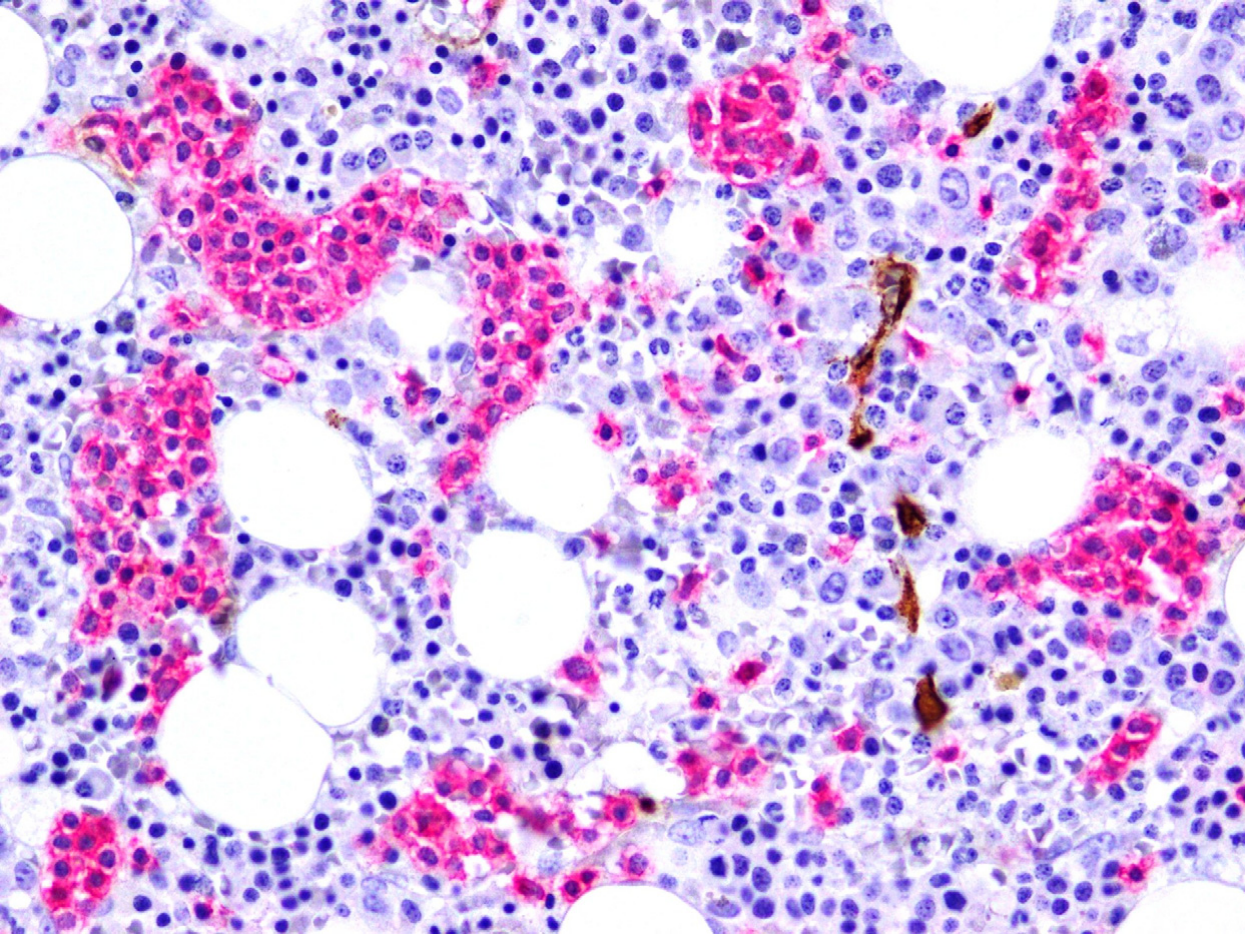
Demonstrates the same, but at x40 magnification.

The patient achieved a partial response to chemotherapy, although unfortunately his disease was refractory to further treatment and progressed within a short timeframe; he received palliative treatment and died comfortably.

Hepatosplenic T‐cell lymphoma is a rare, aggressive form of extranodal lymphoma that originates from cytotoxic T cells, typically *γδ* T‐cell receptor (TCR) type. In addition to hepatosplenic disease, the bone marrow is invariably involved with a characteristic intrasinusoidal pattern of infiltration. Cells are CD3+ and generally *γδ* TCR+ with CD4−, CD5−, CD8−/+ and CD56−/+ 1.

## Authorship

LAB: wrote the submission to Clinical Case Reports. SJ: selected the case, photographed the image, and oversaw the submission process.

## Conflict of Interest

None declared.
